# Use of learner-driven, formative, ad-hoc, prospective assessment of competence in physical therapist clinical education in the United States: a prospective cohort study

**DOI:** 10.3352/jeehp.2023.20.36

**Published:** 2023-12-08

**Authors:** Carey Holleran, Jeffrey Konrad, Barbara Norton, Tamara Burlis, Steven Ambler

**Affiliations:** 1 Program in Physical Therapy, Washington University School of Medicine, St. Louis, MO, USA; 2 Department of Neurology, Washington University School of Medicine, St. Louis, MO, USA; 3 Department of Orthopedic Surgery, Washington University School of Medicine, St. Louis, MO, USA; 4 Department of Internal Medicine, Washington University School of Medicine, St. Louis, MO, USA; Hallym University, Korea

**Keywords:** Cohort studies, Assessment, Competency-based education, Entrustable professional activities, Learning

## Abstract

**Purpose:**

The purpose of this project was to implement a process for learner-driven, formative, prospective, ad-hoc, entrustment assessment in Doctor of Physical Therapy clinical education. Our goals were to develop an innovative entrustment assessment tool, and then explore whether the tool detected (1) differences between learners at different stages of development and (2) differences within learners across the course of a clinical education experience. We also investigated whether there was a relationship between the number of assessments and change in performance.

**Methods:**

A prospective, observational, cohort of clinical instructors (CIs) was recruited to perform learner-driven, formative, ad-hoc, prospective, entrustment assessments. Two entrustable professional activities (EPAs) were used: (1) gather a history and perform an examination and (2) implement and modify the plan of care, as needed. CIs provided a rating on the entrustment scale and provided narrative support for their rating.

**Results:**

Forty-nine learners participated across 4 clinical experiences (CEs), resulting in 453 EPA learner-driven assessments. For both EPAs, statistically significant changes were detected both between learners at different stages of development and within learners across the course of a CE. Improvement within each CE was significantly related to the number of feedback opportunities.

**Conclusion:**

The results of this pilot study provide preliminary support for the use of learner-driven, formative, ad-hoc assessments of competence based on EPAs with a novel entrustment scale. The number of formative assessments requested correlated with change on the EPA scale, suggesting that formative feedback may augment performance improvement.

## Graphical abstract

[Fig f6-jeehp-20-36]

## Introduction

### Background/rationale

Competency-based education (CBE) in the health professions has been described as an outcome-based, developmental approach to instruction and assessment that is aimed at meeting the healthcare needs of society. CBE curricula incorporate a longitudinal, learner-centered approach to instruction, assessment, and promotion [1]. Two goals of CBE are to support learners in (1) achieving competence and (2) developing the skills of the master adaptive learner (MAL), that is, developing the metacognitive processes necessary for self-regulated and lifelong learning [1].

A critical component of self-regulated learning is self-monitoring, the development of which requires explicit feedback [1]. One potentially powerful source of feedback for learning is the authentic clinical environment, known as clinical education. Clinical education provides an indispensable opportunity for learners to be assessed on the task-specific activities of their profession with a clinically meaningful assessment tool. Assessment based on entrustable professional activities (EPAs) is a type of workplace-based assessment that provides information about progression towards clinical competence. EPAs are described in the literature as task-specific activities of a profession in which the task (1) has a clearly defined beginning and end, (2) is specific and focused, (3) is clearly distinguished from other EPAs, (4) reflects work that defines and is essential to a profession, and (5) involves the application and integration of multiple domains of competence [2]. Measurement scales used in prospective entrustment assessments describe the anticipated level or type of supervision a trainee requires for safe and high-quality care in the next patient encounter [3]. Trust has been described as a “central concept for safe and effective healthcare” [3]. Determining trustworthiness of learners is imperative because educators in health professions have the societal obligation to ensure that their graduates can assume the role of independently and safely caring for patients [3].

Assessment across health professions historically has relied on a small number of summative assessments usually performed by a single rater, with limited or no documentation of areas for improvement, and progression has been based on time, not competence [3]. Assessment can be improved by leveraging learning science principles that promote autonomy, self-direction, motivation, self-monitoring, and reflection, all of which help maximize learning and develop MALs [1]. For example, self-monitoring and reflection, critical components of the MAL cycle, are calibrated through repetitive practice in comparing the learner’s self-assessment to that of a credible, external source, described as informed self-assessment [1]. Success in achieving informed self-assessment may be limited by the following: (1) fear of feedback that is contradictory to self-assessment, (2) fear of harming relationships with candid feedback, and (3) barriers within the learning/practice environment [4]. Achievement of informed self-assessment may be facilitated by using assessments that are primarily formative in nature and provide feedback that supports learning [5]. Characteristics of formative feedback include establishing where learners are in their learning and where they are going, as well as explicitly prescribing what they need to do to achieve an outcome [6]. Given that formative feedback is integral to enhancing self-monitoring, reflection, and performance improvement, assessment models that are formative must be developed.

### Objectives

Physical therapy educators need to implement assessment of the essential tasks of a profession in the authentic environment in order to shed light on achievement of competence and to provide information about entrustment to foster best practices in education [7]. The purpose of this project was to implement a process for learner-driven, formative, prospective, ad-hoc entrustment assessment in Doctor of Physical Therapy (DPT) clinical education. Our goals were to develop an innovative entrustment assessment tool that includes an entrustment scale and a structure for providing narrative feedback, and then answer 3 key questions: (1) Does the tool detect differences between learners at different stages of development? (2) Does the tool detect differences within learners across the course of a clinical education experience? (3) Is there a relationship between number of assessments and change in performance?

## Methods

### Ethics statement

This project was deemed exempt by the Institutional Review Board at Washington University in St. Louis (202208009). The data collected was for educational purposes and, therefore, a waiver of consent was granted.

### Study design

**Fig. 1.**
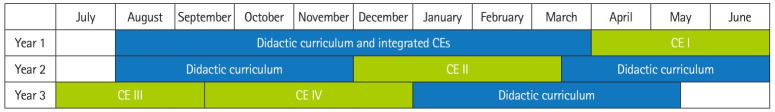
Sequencing of clinical experiences (CEs) across the professional curriculum.

In this prospective cohort study, entrustment scores were collected longitudinally across 4 clinical experiences (CEs) for learners in 2 DPT cohorts, each of which was at a different stage in the developmental continuum. Fig. 1 displays the timing of data capture for each cohort with respect to the curriculum. It was described according to the STROBE (Strengthening the Reporting of Observational Studies in Epidemiology) statement (https://www.strobe-statement.org/).

### Setting

The DPT curriculum at Washington University School of Medicine spans 3 academic years and includes 4 CEs. Graduates are prepared for full licensure to practice in all settings upon graduation. Clinical sites in which the assessments were conducted included the following settings: outpatient orthopedics, neurology, oncology, pelvic health, inpatient rehabilitation, acute care orthopedics, neurology, and pediatrics. After volunteering to participate, separate 30-minute training sessions were held for both clinical instructors and learners on the purpose and the logistics of using the novel EPA tool.

### Participants

**Fig. 2.**
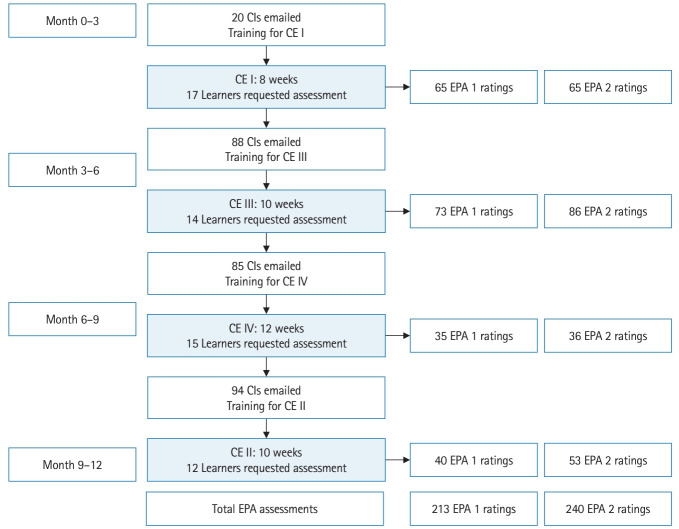
Timeline of recruitment, training for clinical instructors (CIs) and learners, data collection and number of entrustable professional activity (EPA) assessments by clinical experience (CE).

Fig. 2 depicts the process for recruitment. A total of 287 clinical instructors were contacted by email. For learners who were scheduled for CE I, 20 clinical instructors were contacted to trial the logistics of the system in a smaller sample. For the remaining 3 CEs, all clinical instructors were emailed to request their participation. Sixty-nine unique clinical instructors volunteered to participate across 44 clinical sites; 7 of the clinical instructors volunteered for 2 CEs. Sixty-one unique learners were available to request assessment; 6 of the learners were available for 2 CEs. Learners had the option to participate only if their clinical instructors volunteered. No specific inclusion or exclusion criteria were used for this convenience sample, and no volunteers were paid for their participation.

### Variables

The variables included the entrustment scaling score.

### Entrustment Assessment Scale

Prior to this project, EPAs for the physical therapy profession had not been developed, and no articles regarding EPA assessment within DPT clinical education had been published. Thus, the first step was to create a set of EPAs for DPT learners. In 2018, our curriculum renewal writing group reviewed examples of EPAs that were being used in medicine and developed original drafts of EPAs for physical therapists. Next, our faculty’s team of 5 clinical education advisors, all of whom had considerable experience with approaches for assessment in the clinical environment, along with other 4 DPT faculty, who were part of the centralized assessment team for the entire curriculum, began work on developing an entrustment scale. The team reviewed examples of entrustment scales that were being used in undergraduate and graduate medical education [8,9]. They modified the scale to ensure inclusion of learner-centered language, as well as, levels that would capture meaningful gradation across the educational continuum considering expectations for eventual licensure as physical therapists. The team also sent the scale to 2 external site coordinators for clinical education to obtain feedback on clarity of language. The new entrustment scale is depicted in Table 1.

### Data sources/measurement

The timing and frequency of EPA assessments were learner-driven. Clinical instructors were directed to allow learners to choose when and with which patients they would be assessed. The recommendation was for learners to request 1 EPA assessment per week per EPA. Two EPAs were used: (1) gather a history and perform an examination and (2) implement and modify a plan of care, as needed. Clinical instructors were instructed to observe the learner performing the EPA and provide a rating on the scale in response to the following prompt: “Based on your experience with the learner in this patient encounter, at what level would you trust the learner for the next patient encounter?” Clinical instructors were instructed to provide a narrative rationale for their rating in addition to describing what skills were observed, absent, or needed further development. Instructors and learners subsequently co-developed 1–2 goals for ongoing performance improvement. Information from these assessments did not influence any summative decisions. The novel assessment tool was captured via a REDCap survey (Research Electronic Data Capture).

### Bias

Though there was no bias in selecting participants, only learners who were scheduled to train with clinical instructors who volunteered had the opportunity to participate. Learners had the opportunity to participate but were not required to do so. The reasons that learners or clinical instructors chose not to participate are unknown.

### Study size

No study size was estimated. The specific measurement scale developed for this project had never been used and there was no data available on which to base estimates. Only data from voluntarily participating students was included in the analysis.

### Statistical methods

All statistical analyses were performed in the R environment ver. 4.3.1 (R Core Team). All analyses were completed separately for EPA 1 and EPA 2. Kruskal-Wallis analysis of variance was used to detect differences between entrustment scores at different stages of development (i.e., between CEs). A significant Kruskal-Wallis test statistic indicates that the entrustment scores differed across at least 1 CE. Further pairwise comparisons between CEs were performed with the Wilcoxon signed-rank test. Mixed-effects linear regression models were used to detect differences within each CE. This type of model was selected in order to account for the varying numbers of repeated measures within participants. Assessment count, rotation, and patient complexity entered were considered as possible independent variables and were entered in order as part of a step-wise model building process. The corrected Akaike information criterion was used to select the best fitting model. In the final model, the entrustment score was the dependent variable. The assessment count and CE were included as fixed effects and the intercept and assessment count were random effects for each learner. A significant positive fixed effect coefficient from assessment count would confirm that entrustment scores improved over the CEs. Spearman’s rho was used to determine the relationship between the entrustment assessment change score (last score minus first score for each CE) and the number of assessments. The change score and assessment count were determined separately for each CE. A significant positive correlation coefficient would confirm that entrustment score growth was related to the number of assessments. Statistical significance was set at 0.05.

## Results

### Participants

Fig. 2 depicts the timeline and outcomes of recruitment, training for clinical instructors and learners, data collection and number of assessments by CE.

### Main results

**Fig. 3.**
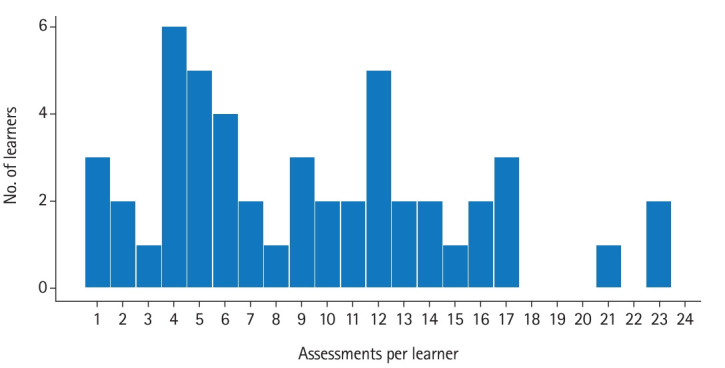
Frequency of number of assessments by learner.

For 49 learners who chose to participate, a total of 453 EPA assessments were collected. Thirteen learners did not request any ad-hoc assessment. Of those learners who requested assessments, the number of assessments per learner ranged from 1 to 23, with a mean of 9.24±5.77 per CE. The number of EPA 1 and EPA 2 assessments for each CE are shown in Fig. 2. Fig. 3 displays the number of learners by number of assessments.

**Fig. 4.**
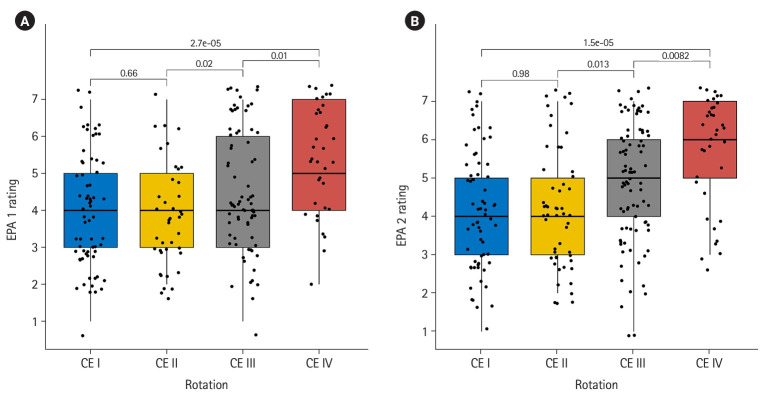
Box plot of entrustable professional activity (EPA) assessments across CEs. (A) EPA 1 and (B) EPA 2.

**Fig. 5.**
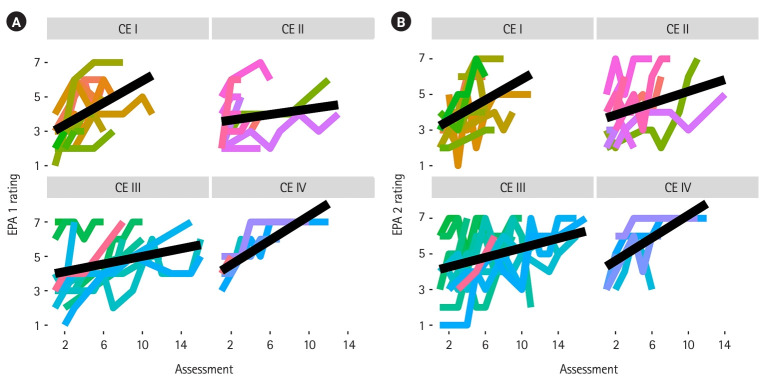
Individual learners’ entrustable professional activity (EPA) assessment plots within clinical experiences (CEs). (A) EPA 1 and (B) EPA 2. Each learner is depicted by a different colored line.

With respect to question 1, the novel EPA assessment tool detected statistically significant differences between the CEs for both EPA 1 (χ^2^=24, P=2.5×10^-5^) and EPA 2 (χ^2^=25.6, P=1.13×10^-5^), which indicates that more advanced learners achieved higher levels of entrustment. Fig. 4A and Fig. 4B display the pairwise changes in entrustment score across the CEs for EPA 1 and EPA 2, respectively. With respect to question 2, linear mixed model regression revealed statistically significant growth across the CEs for both EPAs. The coefficient for assessment count was 0.31 (95% confidence interval [CI], 0.25–0.38; P=6×10^-9^) for EPA 1 and 0.26 (95% CI, 0.20–0.33; P=2×10^-8^) for EPA 2 demonstrating that the scale was able to detect growth within a CE. Fig. 5A and Fig. 5B display individual learner plots across the 4 CEs for EPA 1 and EPA 2, respectively. With respect to question 3, the number of assessments and entrustment score change were positively correlated (EPA 1: ρ=0.47; 95% CI, 0.24–0.66; P=0.0003; EPA 2: ρ=0.54; 95% CI, 0.30–0.72; P=0.00818).

Data file contains raw response items by student per CE are available at Dataset 1.

## Discussion

### Key results

The purpose of this prospective, observational, cohort study was to implement learner-driven, formative, ad-hoc, prospective assessment of competence to facilitate learning and performance improvement within CEs for DPT learners. The novel assessment tool included an entrustment scale and a structure to provide narrative feedback. Data from the novel tool demonstrated differences between learners at different levels, change within each CE, and a correlation between improvement across a CE and number of feedback opportunities.

### Interpretation

Assessment of entrustable professional activities has the potential to provide meaningful guidance for MALs in their ongoing improvement [1], and to offer meaningful information regarding progression towards competence to other stakeholders, such as, health professions educators, health system leaders, and patients [3]. Creating assessment systems that are valid for the purposes of guiding learning and making decisions about competence and entrustment is imperative for safe and effective practice in the health professions; additional data are needed to test the validity of this EPA assessment system fully. Learners were instructed to use ad-hoc, formative assessments to receive feedback that would be important for ongoing performance improvement. Due to the context-dependent nature of competence, variability across assessment events was anticipated. Plots of raw learner assessment data points (Fig. 4A, B) show the variability of ratings within a single learner. Though there is a trend of growth over time, there are peaks and valleys in individual learner scale ratings, with similar trajectories across EPAs. When viewing the initial EPA rating across CEs, though data are not matched between learners, there is variability in initial supervision level despite being at different time points in the professional curriculum. As learners are engaging in new environments, new learning likely is required. This important finding should caution assessors when using single point summative assessments to make high-stakes decisions regarding learner progression, and supports the importance of the ongoing nature of formative assessment across multiple assessors [6]. Further, if learners are using this assessment structure to identify areas for improvement and use this as a learning tool, they should expect that there will be peaks and valleys across a CE. An encouraging finding from this analysis is that the number of times learners engaged in the process of formative assessment was associated with greater changes in their performance. This suggests that creating a formative assessment structure could be influential in learners’ receiving feedback required to promote their entrustability. This study adds to an emerging area of literature focused on assessment of competence in the clinical environment across health professions education.

### Comparison with previous studies

Changes in entrustment scale ratings were larger as learners engaged in more formative assessment. This outcome is consistent with outcomes from studies in other health professions [10], in that it conforms to the predicted trajectory of learning through practice across novice to more advanced learners [10,11]. Thus, these findings provide preliminary support for the validity of this assessment tool.

### Limitations

We did not adjust P-values for multiple comparisons performed on both EPAs because these were distinct tasks, and the purpose of this pilot project was to discover the existence of relationships between the tasks and our novel entrustment tool. Individual learners are not necessarily assessed by multiple clinical instructors. Therefore, we cannot separate a learner’s true ability from the clinical instructors’ perception. Lastly, this study used a convenience sample of clinical instructors who were interested in using this novel EPA tool.

### Generalizability

Use of the tool across a larger sample of learners and clinical instructors is needed to examine generalizability.

### Suggestions

Entrustment scaling alone is unlikely to provide all of the information necessary to calibrate informed self-assessment, which is critical for learning. Further, decisions regarding promotion along the professional learning continuum need to be informed by multiple data points across a program of assessment, including rich narratives captured within the daily tasks of clinical work. Multiple reports across the health professions have identified the critical nature of narratives, with some advocating for narrative descriptions to replace scaling and grading altogether [12]. Clinical performance currently relies on universal summative rating scales, which have not been shown to predict future clinical performance [13]. More work is needed on assessment methods that include high-quality, narrative feedback related to trustworthiness. What is unclear from this analysis is whether or not pairing of narrative feedback with scores from the assessment scale influenced ongoing learning and performance improvement. Subsequent analyses on the quality of feedback and the relationship between quality of feedback and supervision level are necessary and forthcoming.

### Conclusion

This study demonstrates that data from a novel entrustment tool detected differences between learners at different stages of development and differences within learners in a CE. Furthermore, the number of times a learner engaged in formative assessment was positively related to their change in performance. The potential contribution of entrustment-based formative assessment in the authentic clinical environment for promoting learning is an important consideration for health professions educators.

## Figures and Tables

**Figure f6-jeehp-20-36:**
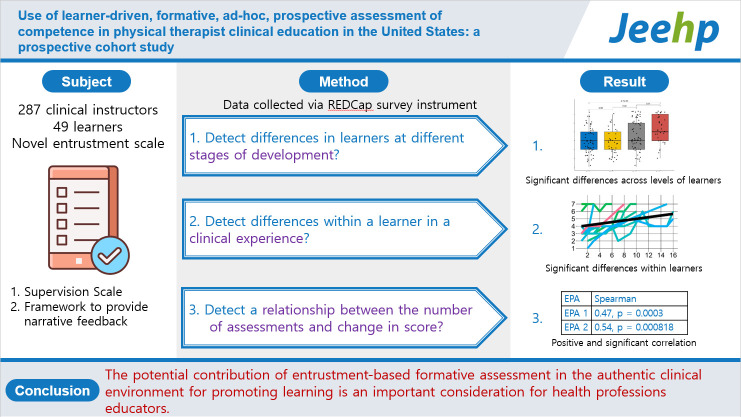


**Table 1.** Entrustment scale adapted for physical therapy clinical education

GME entrustment and supervision scale example	New modified scale
1. Not allowed to practice EPA	1. Learner not trusted to practice EPA
	a. Learner observes supervisor
2. Allowed to practice EPA only under proactive, full supervision	2. Learner trusted to practice EPA coactively
	a. Learner requires continued guidance from supervisor; supervisor in room and participating coactively
	b. Learner requires intermittent, supervisor-initiated guidance; supervisor in room and ready to step in, as needed
3. Allowed to practice EPA only reactive/on-demand supervision	3. Learner trusted to practice EPA with direct supervision
	a. Learner recognizes need for assistance and seeks guidance; supervisor in room and ready to step in, as directed by learner
4. Allowed to practice EPA unsupervised	4. Learner trusted to practice with indirect supervision
	a. Learner’s findings/decisions are double-checked; supervisor available
	b. Learner’s findings/decisions are reviewed retrospectively, then feedback is provided; supervisor available
5. Allowed to supervise others in practice of EPA	5. Learner trusted to practice EPA with mentorship
	a. Learner acts on own; supervisor distantly available

Original graduate medical education 5 level scale. Scale was modified to include 7 levels that would capture meaningful gradation across the educational continuum considering expectations for eventual licensure as physical therapists, and learner-centered language. EPA, entrustable professional activity. GME, graduate medical education.

## Footnotes

**Data availability**
Data files are available from Harvard Dataverse: https://doi.org/10.7910/DVN/5S4WAK Dataset 1. The data file contains raw response items by student per clinical experience.

## Supplementary materials

Supplement 1. Audio recording of the abstract.

## References

[1] Cutrer WB, Miller B, Pusic MV, Mejicano G, Mangrulkar RS, Gruppen LD, Hawkins RE, Skochelak SE, Moore DE (2017). Fostering the development of master adaptive learners: a conceptual model to guide skill acquisition in medical education. Acad Med.

[2] Ten Cate O, Taylor DR (2021). The recommended description of an entrustable professional activity: AMEE guide no. 140. Med Teach.

[3] Ten Cate O, Hart D, Ankel F, Busari J, Englander R, Glasgow N, Holmboe E, Iobst W, Lovell E, Snell LS, Touchie C, Van Melle E, Wycliffe-Jones K, International Competency-Based Medical Education Collaborators (2016). Entrustment decision making in clinical training. Acad Med.

[4] Mann K, van der Vleuten C, Eva K, Armson H, Chesluk B, Dornan T, Holmboe E, Lockyer J, Loney E, Sargeant J (2011). Tensions in informed self-assessment: how the desire for feedback and reticence to collect and use it can conflict. Acad Med.

[5] Black P, Wiliam D (2009). Developing the theory of formative assessment. Educ Assess Eval Account.

[6] Lockyer J, Carraccio C, Chan MK, Hart D, Smee S, Touchie C, Holmboe ES, Frank JR, ICBME Collaborators (2017). Core principles of assessment in competency-based medical education. Med Teach.

[7] Jensen GM, Jette DU, Timmerberg JF, Chesbro SB, Dole RL, Kapasi Z, Lotshaw A (2022). Competency-based education in physical therapy: developing a framework for education research. J Phys Ther Educ.

[8] Chen HC, van den Broek WE, ten Cate O (2015). The case for use of entrustable professional activities in undergraduate medical education. Acad Med.

[9] Rekman J, Gofton W, Dudek N, Gofton T, Hamstra SJ (2016). Entrustability scales: outlining their usefulness for competency-based clinical assessment. Acad Med.

[10] Violato C, Cullen MJ, Englander R, Murray KE, Hobday PM, Borman-Shoap E, Ersan O (2021). Validity evidence for assessing entrustable professional activities during undergraduate medical education. Acad Med.

[11] Thompson BM, Rogers JC (2008). Exploring the learning curve in medical education: using self-assessment as a measure of learning. Acad Med.

[12] Hanson JL, Rosenberg AA, Lane JL (2013). Narrative descriptions should replace grades and numerical ratings for clinical performance in medical education in the United States. Front Psychol.

[13] White CB, Fantone JC (2010). Pass-fail grading: laying the foundation for self-regulated learning. Adv Health Sci Educ Theory Pract.

